# Validation of the Indonesian version of the Safety Attitudes Questionnaire: A Rasch analysis

**DOI:** 10.1371/journal.pone.0215128

**Published:** 2019-04-10

**Authors:** Evi Ningrum, Sue Evans, Sze-Ee Soh

**Affiliations:** 1 Department of Epidemiology and Preventive Medicine, Monash University, Melbourne, VIC, Australia; 2 School of Nursing, Brawijaya University, Malang, East Java, Indonesia; 3 Department of Physiotherapy, Monash University, Melbourne, VIC, Australia; Universidad de Murcia, SPAIN

## Abstract

**Introduction:**

Safety climate, which provides a snapshot of safety culture, is rarely measured in Indonesian healthcare organisations because there are no validated surveys that can be administered in its native language, Bahasa Indonesia. The objectives of this study were to translate and linguistically adapt the Safety Attitudes Questionnaire into Bahasa Indonesia, and investigate the internal construct validity and reliability of the translated survey.

**Methods:**

The Safety Attitudes Questionnaire was translated into Indonesian language through forward and backward translation. The internal construct validity and reliability of the translated survey was assessed using Rasch analysis which examines overall model fit, unidimensionality, response format, targeting, internal consistency reliability and item bias.

**Results:**

A total of 279 nurses (response rate 82%) completed the Indonesian version of the Safety Attitudes Questionnaire. Most respondents were Division 2 registered nurses (*n* = 209; 75%), female (*n* = 174; 62%), and aged less than 30 years (*n* = 187; 67%). All six domains of the Indonesian version of the Safety Attitudes Questionnaire demonstrated unidimensionality (*t*-test less than 0.05 threshold value). However, suboptimal targeting (ceiling effect) was observed in all domains, and had at least one misfitting item (item fit residual beyond ±2.5) Item bias was also evident in most domains.

**Conclusion:**

This study has translated and validated an Indonesian version of the Safety Attitudes Questionnaire for the first time. Whilst there was general support to sum items to obtain domain scores, further work is required to refine the response options as well as the wording and number of items in this survey to improve its overall measurement properties.

## Introduction

Safety culture is an aspect of organisational culture that refers to the views, perceptions and actions of personnel within an organisation towards safety management and policy [1, 2]. Due to the complexities of measuring culture [3], safety climate surveys are often used to provide a snapshot of the culture of safety within an organisation [4]. There is emerging evidence demonstrating that low levels of safety climate may lead to poor safety outcomes and adverse events, such as increased length of stay [5], higher postoperative mortality rates [6] and higher 30-day readmission rates [7]. Adverse events in Indonesian hospitals have been reported to be as high as 171 cases among 285 admissions in Jakarta [8]. This suggests that there may be issues around safety climate in these hospitals. Thus, measuring safety climate in this setting may be helpful to guide the design and delivery of appropriate quality improvement programmes to improve patient safety.

A variety of instruments can be used to measure safety climate such as the Hospital Survey on Patient Safety Culture (HSOPSC) [9], the Safety Organising Scale (SOS) [10] and the Safety Attitudes Questionnaire (SAQ) [11]. The HSOPSC has previously been translated into Bahasa, Indonesia’s native language, to measure safety climate in Indonesian acute hospitals [12, 13]. However, content validity of the translated HSOPSC was not examined and it demonstrated poor test-retest reliability [12, 13] making it an inappropriate instrument to quantify safety climate in Indonesian hospitals.

The SAQ (S1 Questionnaire) is currently the most widely used safety climate tool and has the most evidence to support its validity and reliability [11, 14–16]. Confirmatory factor analysis (CFA) has shown that a six-factor structure resulted in a satisfactory model fit for the majority of fit indices [11]. Translated versions of the SAQ in Dutch [17], Norwegian [18], Chinese [19], Swedish [20, 21] and Italian [22] have also demonstrated good validity and reliability, with good model fit when the factor structure was examined using CFA [18, 19, 21]. Given the lack of a valid instrument to assess safety climate in Indonesia, and the success of others in translating and validating the SAQ, the primary aim of this study was to translate and linguistically adapt the SAQ into the Indonesian language (SAQ-INA). In order to ensure that the SAQ-INA can be used to quantify safety climate in Indonesian hospitals, Rasch analysis was used to evaluate its key measurement properties and internal construct validity.

## Materials and methods

### Design, settings and participants

A cross-sectional study design was adopted to assess the internal construct validity of the SAQ-INA. Study participants were licenced nurses who performed direct clinical care to patients in all Emergency Department (ED) and inpatient units at the Ngudi Waluyo public hospital in Blitar, East Java, Indonesia and the Wava Husada private hospital in Malang, East Java, Indonesia. Both hospitals are medium-sized sized hospitals with approximately 120 to 170 beds, and employ approximately 250 and 300 nurses respectively. Nurses working in both hospitals may hold either a bachelor degree (division 1 registered nurses) or a diploma degree (division 2 registered nurse).

To be eligible to participate in this study, nurses must have worked in either hospital for at least 7.5 hours a week, two months before the survey was administered [16]. We excluded nurses who worked less than 7.5 hours a week because they may be less aware of the culture on the ward and/or hospital [16]. Prior to data collection, an explanatory statement was distributed along with the survey to potential participants by research assistants and the principal researcher (EN). Distribution of surveys generally occurred at the time of shift change to facilitate immediate completion of the survey. The explanatory statement emphasised to the nurses that participation in the study was voluntary and that completion of the survey denoted that they had agreed to participate (implied consent). Ethical approval was obtained from the Human Research Ethic Committee at Monash University (MUHREC 2016–1409).

### The Safety Attitudes Questionnaire

The SAQ which was derived from a tool used in aviation, assesses six safety-related domains, namely teamwork climate (6 items), safety climate (7 items), perceptions of management (5 items), job satisfaction (5 items), working conditions (4 items) and stress recognition (4 items) [11]. Five items in the perceptions of management domain assess the ward and hospital units separately [11]. As such, the SAQ consists of a total of 41 items. Each SAQ item is measured using a five point Likert scale that ranges from strongly disagree to strongly agree with a neutral mid-point category. Higher scores on the SAQ reflect higher levels of safety climate. Benchmarking data is available for the SAQ which facilitates evaluation of safety climate data between different wards and hospitals [11].

### Translation into Bahasa Indonesian

The SAQ was translated by a professional translator to Bahasa Indonesia and back translated by another independent professional translator blinded to the English version of the survey, to achieve a cross-cultural and conceptual equivalent instrument [23]. The same response categories (i.e. five-point Likert scale) was used for this translated version of the SAQ and the final version (S2 Questionnaire) was pilot tested with a representative sample of 10 Indonesian nurses to assess for clarity, wording appropriateness, and acceptability. No issues or corrections were identified from this process.

## Rasch analysis

Rasch analysis was used to examine the internal construct validity of the SAQ-INA in this sample of Indonesian nurses. There is a growing recognition that modern psychometric approaches based on item response theory (e.g. Rasch analysis) has advantages over classical test theory approaches including CFA [24]. Specifically, Rasch analysis allowed the following measurement properties of the SAQ-INA to be assessed:

1. Unidimensionality—whether items in each domain measured the same underlying construct;
2. Response thresholds—whether nurses were able to discriminate between the different options of the five-point Likert scale;
3. Internal consistency reliability–whether items in each SAQ-INA domain can differentiate varying levels of safety climate;
4. Targeting–whether items in each domain had floor or ceiling effects; and
5. Differential item functioning (DIF)–whether items in each domain were biased towards specific groups despite having equal levels of safety climate [16, 25–27].

As a six-factor structure of the SAQ has been confirmed in previous studies [11, 18, 19, 21], we focused on examining the unidimensionality for each SAQ-INA domain to determine whether the items can be summed to generate an overall domain score.

### Statistical analysis

Overall model fit was examined to determine whether the SAQ-INA data met the expectations of the Rasch measurement model. This was assessed using the χ^**2**^ item-trait interaction statistic, where a non-significant value (*p*>0.05) indicated that the observed model met the expectations of the Rasch model [26]. Model fit was also assessed by examining item-person interaction statistics, where a residual standard deviation (SD) of ≤1.5 indicated satisfactory fit, and the residual fit statistics of individual item- and person-fit where values between -2.5 and 2.5 indicated adequate fit [26]. Details of additional measurement properties examined in Rasch analysis apart from overall model fit are described in Table 1. The SAQ-INA data were analysed using SPSS version 23 and the Rasch analysis was conducted using the RUMM 2030 package with a partial credit model (RUMM Laboratory Pty. Ltd., Perth, Australia).

**Table 1:** Measurement properties and criteria assessed in the Rasch analysis.

Measurement properties	Purpose	Statistical test	Measurement criteria
**Unidimensionality and local dependency**	To assess whether items in each SAQ-INA domain measured the same underlying construct	• PCA of residual • Equating t-test • Binomial dimensionality test	Identify two subsets of items from the first factor extracted by PCA [26]. Compare person estimates from the two different subsets using independent *t*-tests, where *p*<0.05 indicates the domain is unidimensional. If *p*>0.05, the value of 5% obtained from a binomial test of proportions should be included in the 95% CI [26].
	To assess whether the response to an item is dependent on the response to another item (i.e. local dependency which is an element of unidimensionality).	• Person-item residual correlation	A person-item residual correlation value of >0.2 indicated the presence of local dependency [26].
**Response format (thresholds)**	To assess whether participants could discriminate between the different response options of the five-point Likert scale.	• Threshold map • Category probability curves	Visually inspect the pattern of response options for each SAQ-INA item. Thresholds are considered to be ordered when each response option is the most likely response at some point along the location continuum [26].
**Targeting**	To assess whether items in each SAQ-INA domain had floor or ceiling effects.	• Mean location score • Person-item distribution threshold distribution map	A mean logit score of zero indicates a well-targeted scale i.e. no floor or ceiling effects [26]/
**Internal consistency reliability**	To assess whether items in each SAQ-INA domain can differentiate varying levels of safety climate.	• Person separation index	A value of >0.7 suggests that the SAQ-INA items has good internal consistency reliability [16, 26].
**Item bias**	To assess whether items in each SAQ-INA domain were biased towards specific groups (e.g. public versus private hospitals).	• Differential item functioning (DIF)	Significant main (uniform DIF) and interaction (non-uniform DIF) effects (*p*<0.05) indicates that an item may be biased for different groups e.g. public versus private hospitals [26].

SAQ-INA: Safety Attitudes Questionnaire–Indonesian version; PCA: Principal Component Analysis; CI: Confidence interval

### Sample size

The sample size for a Rasch analysis required to obtain an appropriate degree of precision depends on whether items have been appropriately targeted to participants in the sample [16, 26]. If a scale is well targeted with minimal standard error, a sample size of 108 is sufficient; if not, a sample size of at least 243 will be required [16, 28].

## Results

### Participant characteristics

A total of 340 surveys were distributed to nurses working in 17 wards across two Indonesian hospitals. Of these, 279 surveys were returned giving an overall response rate of 82%. Most participants were female nurses (*n* = 174; 62%) aged less than 30 years old (*n* = 187; 67%). The majority of nurses (*n* = 209; 75%) were employed as Division 2 Registered Nurses, which means that they had a Diploma of Nursing obtained from a nursing vocational college. Most nurses were permanent non-government staff (*n* = 177; 63%) where their salaries are paid by the hospital not by the Indonesian government.

### Overall fit to the Rasch measurement model

Results of the Rasch analysis for the SAQ-INA are described in Table 2. Inspection of the total χ^2^ item-trait interaction statistics showed that all SAQ-INA domains had some degree of misfit between the data and the model (χ^**2**^ = 41.8–199; *p*<0.001). Person misfit was observed in the perception of hospital management domain with a fit residual mean of -0.7 and SD of 1.64. Item fit residual SD values ranged from 1.7 to 4.9 in all SAQ-INA domains, suggesting that item misfit was the most likely contributor to lack of overall model fit.

**Table 2:** Overall model fit for the SAQ-INA domains^a^.

	SAQ-INA domains						
	Teamwork climate	Safety Climate	Job satisfaction	Stress recognition	Perception of ward management	Perception of hospital management	Working condition
**Items**							
**Fit Residual mean^b^**	-0.54	0.06	-1.28	0.03	0.08	0.29	-0.26
**Fit residual SD^c^**	2.21	2.55	2.03	1.76	4.12	4.92	1.73
**Persons**							
**Fit Residual mean^b^**	-0.69	-0.61	-0.89	-0.47	-0.65	-0.68	-0.82
**Fit Residual SD^c^**	1.39	1.47	1.53	1.02	1.49	1.64	1.52
**Total item-trait interaction statistics**							
**Total Item χ^2^**	41.48	75.91	32.00	30.39	199.12	193.78	27.32
**d*f***	18	21	15	12	15	15	12
***p-*value^d^**	0.001	<0.001	0.006	0.002	<0.001	<0.001	0.006
**Person separation index^e^**	0.67	0.73	0.73	0.78	0.68	0.74	0.65

SAQ-INA: Safety Attitudes Questionnaire–Indonesian version; SD: standard deviation; d*f*: degress of freedom

^a^Rasch model is analysed using the RUMM2030 (RUMM Laboratory Pty. Ltd., Perth, Australia)

^b^ Values should be close to 0 [26]

^c^ Values should be close to 1 [26]

^d^ Significant values (*p*<0.005) indicates overall misfit to the Rasch model

^e^ Values between 0.6–0.7 indicates acceptable to good internal consistency reliability [26]

Individual item fit analysis demonstrated that each SAQ-INA domain had at least one misfitting item, except for the stress recognition and working conditions domains. As shown in Table 3, five SAQ-INA items had fit residual values that were less than negative 2.5, which indicates possible item redundancy because the responses to these items were predictable. In contrast, four items had fit residual values greater than positive 2.5, which suggests low levels of discrimination that may compromise how well these items measure the underlying construct [26].

**Table 3:** Individual item misfit in each the SAQ-INA domain.

SAQ-INA domain	Identified item misfit	Fit residual value ^a^	χ^2^ statistic ^b^	Interpretation
**Teamwork climate** **(*p<*0.008)**	SAQ-INA item 2	3.92	<0.001	Low levels of discrimination
**Safety climate** **(*p*<0.007)**	SAQ-INA item 8	-3.12	0.004	Item redundancy
	SAQ-INA item 11	4.56	<0.001	Low levels of discrimination
**Job satisfaction** ***(p<*0.01)**	SAQ-INA item 17	-3.61	0.04	Item redundancy
	SAQ-INA item 18	-2.56	0.04	Item redundancy
**Perception of ward Management** **(*p*<0.01)**	SAQ-INA item 25	7.35	<0.001	Low levels of discrimination
	SAQ-INA item 26	-2.64	<0.001	Item redundancy
**Perception of hospital management** **(*p<*0.01)**	SAQ-INA item 25	9.08	<0.001	Low levels of discrimination
	SAQ-INA item 26	-2.62	0.001	Item redundancy

SAQ-INA: Safety Attitudes Questionnaire–Indonesian version

^a^ Values below -2.5 and above 2.5 indicates item misfit [26]

^b^ Significant values (*p*<0.005) indicates significant item misfit [26]

### Response thresholds

Given that a number of misfitting items were observed, the pattern of thresholds for each domain were examined to determine whether disordering may have contributed to the misfit. Apart from the stress recognition domain, the remaining SAQ-INA domains had at least one disordered threshold (S1 Fig). Inspection of category probability curves also indicated that participants were not able to differentiate consistently between the different options of the five-point Likert scale that was used. Specifically, they had difficulty differentiating between the ‘disagree’ and ‘neutral’ options, as well as the ‘neutral’ and ‘agree’ options. As it was the neutral midpoint category that appeared to contribute to the disordered thresholds, we were unable to collapse the response options. It was unclear whether participants would have selected either the ‘disagree’ or ‘agree’ option if the ‘neutral’ category was not available.

### Unidimensionality and local dependency

All six SAQ-INA domains were found to be unidimensional, which supports summation of items in each domain [26]. Unidimensionality was supported by Principal Component Analysis (PCA) on the residuals (paired t-tests <0.05) except for the “perceptions of ward management” and “working conditions” domain. However, further inspection of the 95% Confidence Interval (CI) for these two domains included the value of 0.05 which indicates that the items were measuring the same underlying construct. Two items in the “perception of ward management” domain (items 26 and 27) were also observed to be locally dependent. This means that participants answered item 26 in the same manner as item 27.

### Targeting

Apart from the stress recognition (mean logit score 0.2) and perceptions of hospital management (mean logit score 0.4) domains, the remaining domains of the SAQ-INA were poorly targeted (mean logit score range 1.0 to 3.5). The person-item distribution graphs for each SAQ-INA domain (S2 Fig) indicate that there were ceiling effects for all domains, with an absence of items at higher levels of safety climate. There were also no items measuring participants’ level of safety climate for most domains at the one to three logit point, which reflects a mid to high SAQ-INA domain score.

### Internal consistency reliability

Most domains of the SAQ-INA had reasonably good internal consistency reliability supported by Person Separation Index (PSI) values ranging from 0.7 to 0.8 (Table 2). The teamwork climate, perception of ward management and working condition domains had PSI values lower than 0.7. This was considered to be acceptable because the presence of disordered thresholds may have contributed to the lower PSI value in the teamwork climate domain, while the lower value for the perception of ward management domain was likely due to the presence of locally dependent items. Likewise, the small number of items (*n* = 4) in the working conditions domain may have resulted in a PSI value of 0.65 [16, 26, 29].

### Item bias

Differential item functioning was used to identify whether nurses responded differently to items in each SAQ-INA domain according to their age group, level of experience, hospital type and ward class. Uniform DIF, where nurses responded differently in a consistent manner were observed in several items (Table 4). Specifically, nurses who worked in public hospitals, who had less working experience and who worked in lower class wards appeared to respond differently to these items. A significant interaction effect (*p*<0.05) was observed for item 10, indicating that nurses who worked in a public hospital responded differently compared to those in the private sector. These responses, however, were not consistent and may contribute to model misfit [26].

**Table 4:** Item bias across person factors.

Person factors	SAQ-INA Items with uniform DIF	
Age groups ^a^	Perception of ward management	24 (ward)
	Working condition	29 and 31
Level of experience ^b^	Safety climate	9 and 13
	Job satisfaction	18
	Working condition	29
Ward class ^c^	Safety climate	7
Hospital type ^d^	Teamwork climate	2
	Perception of hospital management	24, 25 and 26 (hospital)
	Working condition	29, 31 and 32

SAQ-INA: Safety Attitudes Questionnaire–Indonesian version; DIF: Differential item functioning

^a^ ≤30 years and >30 years old

^b^ ≤ 5 years and >5 years experience

^c^ lower class, upper class and no class

^d^ public hospital and private hospital.

## Discussion

In this study, we translated the SAQ into the Indonesian language (SAQ-INA) and examined its capacity to adequately assess safety climate in Indonesian health services using Rasch analysis. Our results demonstrate that all six domains were unidimensional, and that it is appropriate to sum individual items to obtain domain scores. However, there were some notable issues with the instrument; notably ceiling effects were observed for all domains and there were misfitting items. Nevertheless, this is the first study to have translated and validated the SAQ into the Indonesian language using Rasch analysis. The strength of the Rasch measurement model is the ability to identify measurement issues of the SAQ-INA such as response thresholds or item bias by comparing the response patterns of individuals to the entire sample [27, 30]. Thus, our findings can be used to inform the refinement of the SAQ-INA in future studies so that it can accurately measure the level of safety climate in Indonesian hospitals.

The presence of disordered thresholds, particularly in the teamwork and safety climate domains, may have affected the fit of the overall model and individual items [31]. The option of the ‘neutral’ category may have contributed to the disordered thresholds as participants had difficulty differentiating between this option and the ‘disagree’ or ‘agree’ options [16]. This has been identified in previous validation studies of the SAQ [16] and as we were unable to rescore these categories, there is a need to refine the response categories of the SAQ-INA. In particular, future studies should examine whether changing the response categories to a unipolar scale (e.g. not all agree, agree, strongly agree) can improve the overall model fit of the SAQ-INA to the Rasch measurement model.

The ceiling effects observed in this study suggests that the SAQ-INA may have limited ability to capture nurses with high levels of safety climate. This is consistent with findings from previous Rasch validation of the SAQ [16] and highlights the need to refine the wording of existing items in order to improve the measurement of safety climate particularly at the mid-to-high end of the scale. This includes investigating whether the addition of items from other safety climate questionnaires such as the HSOPSC [9] or the SOS [10] may improve the overall targeting of the SAQ-INA. By increasing the number of items within each domain and developing an item bank, it may be possible to measure safety climate with better precision and monitor changes over time [32, 33].

Results of the Rasch analysis indicated the presence of misfitting items in most SAQ-INA domains, with four items displaying low levels of discrimination (i.e. fit residual >2.5). Whilst this may be due to the presence of disordered thresholds, it may be beneficial for future studies to examine whether removing these items would improve the measurement properties of the SAQ-INA. Of particular interest is whether it would improve the internal construct validity of the teamwork and safety climate domains as both domains had one item each with a low level of discrimination. It is also important to note that even though no misfitting items were observed in the stress recognition domain, previous studies have found that this domain performs differently compared to other SAQ domains [16, 29, 34]. Items in this domain tend to assess personal reactions rather than self-behaviour which other domains tend to measure [29]. This means that items in the stress recognition domain may disrupt the underlying constructs of the SAQ-INA [34].

Our study identified that nurses working in public and private hospitals who have the same level of safety climate appear to respond differently to item 2 in the teamwork climate domain. Similarly, nurses with higher levels of experience responded differently to items 9 and 13 in the safety climate domain. This items bias suggests that some items in the SAQ-INA may not measure their respective underlying construct [35]. Whilst removing these items could improve overall model fit, this approach is not recommended because it will decrease the reliability of the scale [35]. Further investigation of how the SAQ-INA performs in other Indonesian nurses and health professional groups is warranted.

Despite the excellent response rate (82%), some limitations of this study need to be considered. Firstly, the generalisability of this study is limited to only nurses and our sample may not be representative of other health professionals such as physicians, pharmacists and other allied health staff. Secondly, as we only examined the level of safety climate in two hospitals, we are unable to generalise our result to nurses working in other Indonesian hospitals. Given that model fit may be influenced by sample characteristics, a larger, more diverse sample may have achieved better measurement precision.

## Conclusion

This is the first study to have translated and examined the internal construct validity and reliability of an Indonesian version of the SAQ using Rasch analysis. Whilst it is appropriate to sum items to obtain scores for individual domains such as teamwork and safety climate, further studies are required to validate the SAQ-INA in larger samples with a refined response format and item wording so that it can be used to reliably measure the level of safety climate in Indonesian hospitals.

## Supporting information

S1 Questionnaire The Safety Attitudes Questionnaire. Reprinted from Sexton et al [11] under a CC BY license, with permission from The University of Texas at Austin, original copyright 2004.
(DOCX)

S2 Questionnaire Indonesian version of the Safety Attitudes Questionnaire (SAQ-INA). (DOCX)

S1 Fig Thresholds maps for the SAQ-INA domains. (DOCX)

S2 Fig Person-item thresholds distribution for all SAQ-INA items. (DOCX)
