# Supplementary material for: Validation of the Indonesian version of the Safety Attitudes Questionnaire: A Rasch analysis
Source: PLoS One. 2019 Apr 10;14(4):e0215128. doi: 10.1371/journal.pone.0215128 (PMC6457536; doi:10.1371/journal.pone.0215128)
Supplement: S1 Questionnaire — Reprinted from Sexton et al [11] under a CC BY license, with permission from The University of Texas at Austin, original copyright 2004. (DOCX) [file pone.0215128.s001.docx]

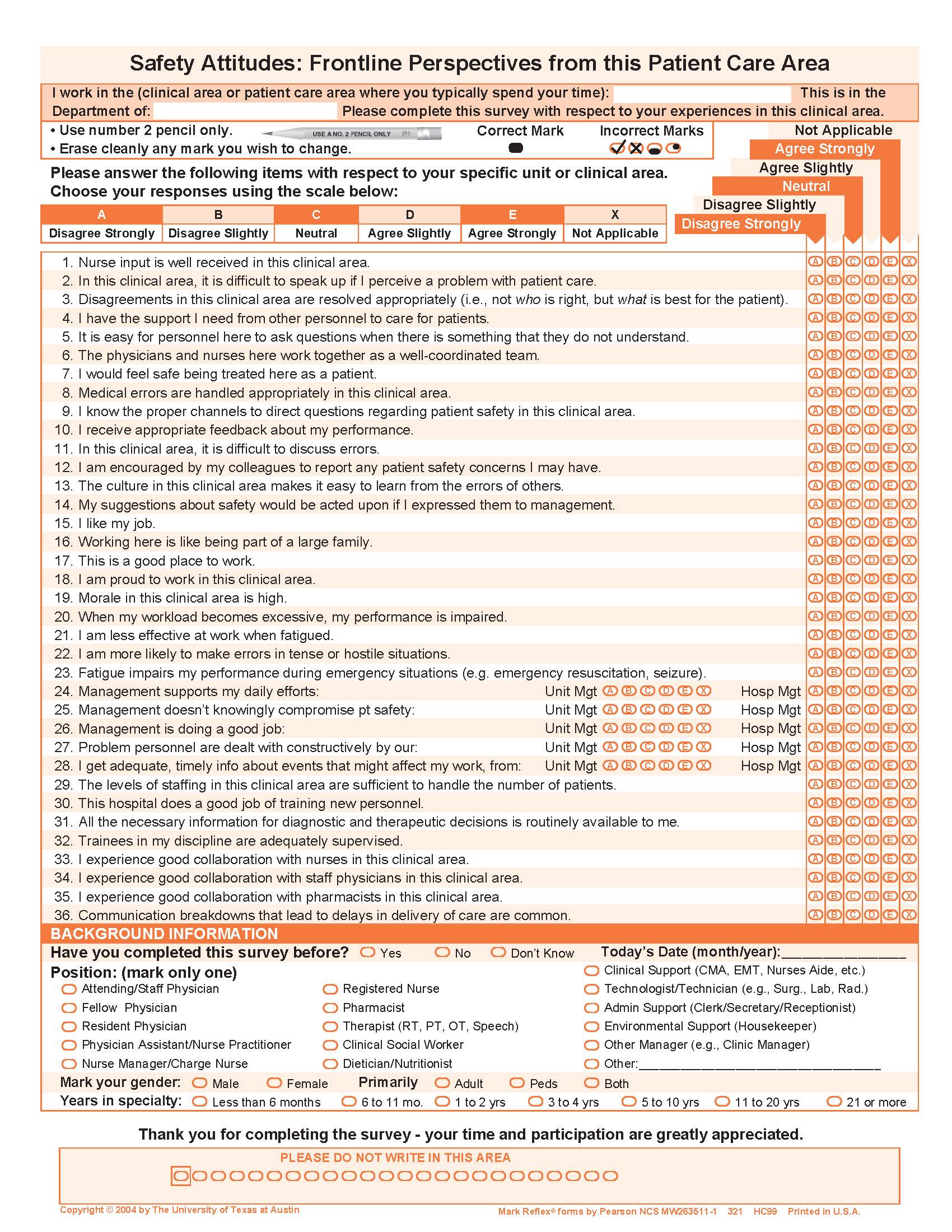


S1 Questionnaire. The Safety Attitudes Questionnaire. Reprinted from Sexton et al [11] under a CC BY license, with permission from The University of Texas at Austin, original copyright 2004.
