# Supplementary material for: Validation of the Indonesian version of the Safety Attitudes Questionnaire: A Rasch analysis
Source: PLoS One. 2019 Apr 10;14(4):e0215128. doi: 10.1371/journal.pone.0215128 (PMC6457536; doi:10.1371/journal.pone.0215128)
Supplement: S2 Questionnaire — (DOCX) [file pone.0215128.s002.docx]

Silahkan lingkari tanggapan yang paling cocok dengan persepsi/pengalaman Anda mengenai keadaan keselamatan **di bangsal dimana Anda paling sering kerja.**

| **Silahkan jawab pernyataan berikut sesuai dengan bangsal Anda menggunakan skala di bawah.** | | | | | | | | | | | | | | |  | | Sangat setuju▼ | | | | | | | | | |  |
| --- | --- | --- | --- | --- | --- | --- | --- | --- | --- | --- | --- | --- | --- | --- | --- | --- | --- | --- | --- | --- | --- | --- | --- | --- | --- | --- | --- |
| A | B | C | D | E | | | | |  | | |  | | |  | | Setuju▼ | | | | | | | | |  |  |
| Sangat tidak setuju | Tidak setuju | Netral | Setuju | Sangat setuju | | | | |  | | |  | | | Netral▼ | | | | | | | |  |  | | |  |
|  | | | | | | | | | | | | Tidak setuju▼ | | | | | | | | |  | |  |  | | |  |
|  | | | | | | | | | Sangat tidak setuju▼ | | | | | | | | |  | | | |  |  | |  | |  |
| Masukan perawat diterima dengan baik di bangsal ini. | | | | | | | | | | | | | | | | A | | | B | | C | | D | E | | |  |
| Di bangsal ini, sukar untuk berbicara jika saya melihat masalah dengan perawatan pasien. | | | | | | | | | | | | | | | | A | | | B | | C | | D | E | | |  |
| Perselisihan pendapat dalam bangsal ini diselesaikan dengan tepat (yaitu bukannya *siapa* yang benar, tetapi *apa* yang terbaik bagi pasien). | | | | | | | | | | | | | | | | A | | | B | | C | | D | E | | |  |
| Saya mendapat bantuan yang saya perlukan dari staf lain untuk perawatan pasien. | | | | | | | | | | | | | | | | A | | | B | | C | | D | E | | |  |
| Mudah bagi pegawai di sini untuk bertanya jika ada sesuatu yang mereka tidak mengerti | | | | | | | | | | | | | | | | A | | | B | | C | | D | E | | |  |
| Dokter, perawat dan staf kesehatan sejenis dalam bangsal ini bekerjasama sebagai tim yang terkoordinasi dengan baik | | | | | | | | | | | | | | | | A | | | B | | C | | D | E | | |  |
| Saya akan merasa aman dirawat di sini sebagai pasien.. | | | | | | | | | | | | | | | | A | | | B | | C | | D | E | | |  |
| Kesalahan medis ditangani dengan tepat dalam bangsal ini. | | | | | | | | | | | | | | | | A | | | B | | C | | D | E | | |  |
| Saya tahu jalur yang tepat untuk mengarahkan pertanyaan mengenai keselamatan pasien di bangsal ini. | | | | | | | | | | | | | | | | A | | | B | | C | | D | E | | |  |
| Saya menerima umpan balik yang sesuai mengenai prestasi saya. | | | | | | | | | | | | | | | | A | | | B | | C | | D | E | | |  |
| Di bangsal ini, sukar untuk membicarakan kesalahan. | | | | | | | | | | | | | | | | A | | | B | | C | | D | E | | |  |
| Budaya dalam bangsal ini membuat staff mudah belajar dari kesalahan orang lain. | | | | | | | | | | | | | | | | A | | | B | | C | | D | E | | |  |
| Saya sangat didorong oleh rekan saya untuk melaporkan kekhawatiran yang saya punya mengenai keselamatan pasien. | | | | | | | | | | | | | | | | A | | | B | | C | | D | E | | |  |
| Saran saya mengenai keselamatan akan ditindaklanjuti jika saya laporkan kepada manajer di bangsal ini. | | | | | | | | | | | | | | | | A | | | B | | C | | D | E | | |  |
| Saya suka pekerjaan saya. | | | | | | | | | | | | | | | | A | | | B | | C | | D | E | | |  |
| Bekerja di bangsal ini seperti menjadi bagian dari suatu keluarga besar. | | | | | | | | | | | | | | | | A | | | B | | C | | D | E | | |  |
| Bangsal ini tempat kerja yang baik. | | | | | | | | | | | | | | | | A | | | B | | C | | D | E | | |  |
| Saya bangga bekerja di bangsal ini. | | | | | | | | | | | | | | | | A | | | B | | C | | D | E | | |  |
| Semangat kerja antara staf di bangsal ini sangat tinggi. | | | | | | | | | | | | | | | | A | | | B | | C | | D | E | | |  |
| Apabila beban kerja saya menjadi berlebihan, kinerja saya terganggu. | | | | | | | | | | | | | | | | A | | | B | | C | | D | E | | |  |
| Saya kurang efektif di tempat kerja jika kelelahan. | | | | | | | | | | | | | | | | A | | | B | | C | | D | E | | |  |
| Saya akan lebih cenderung membuat kesalahan dalam suasana tegang dan berseteru. | | | | | | | | | | | | | | | | A | | | B | | C | | D | E | | |  |
| Kelelahan mengganggu kinerja saya pada waktu keadaan darurat (resasitasi darurat, serangan jantung tiba-tiba). | | | | | | | | | | | | | | | | A | | | B | | C | | D | E | | |  |
| Manajemen membantu usaha harian saya: | | | | Mgt  Bangsal | A | B | C | | D | E | | | | Mgt  R.S | | A | | | B | | C | | D | E | | |  |
| Manajemen tidak secara tahu mengompromi keselamatan pasien: | | | | Mgt  Bangsal | A | B | C | | D | E | | | | Mgt  R.S | | A | | | B | | C | | D | E | | |  |
| Manajemen menjalankan pekerjaannya dengan baik: | | | | Mgt  Bangsal | A | B | C | | D | E | | | | Mgt  R.S | | A | | | B | | C | | D | E | | |  |
| Masalah staf diselesaikan secara konstruktif oleh manager kami(bangsal/RS) | | | | Mgt  Bangsal | A | B | C | D | | | E | | Mgt  R.S | | | A | | | | B | C | | D | E | | | |
| Saya mendapat informasi yang cukup, tepat waktu mengenai peristiwa yang mungkin mengganggu pekerjaan saya dari:: | | | | Mgt  Bangsal | A | B | C | | D | | E | | Mgt  R.S | | | A | | B | | | C | | D | | | E |  |
| Tingkat jumlah staf dalam bangsal ini cukup untuk menangani jumlah pasien. | | | | | | | | | | | | | | | | A | | | B | | C | | D | E | | |  |
| Rumah sakit ini melalukan pekerjaannya melatih staf baru dengan baik. | | | | | | | | | | | | | | | | A | | | B | | C | | D | E | | |  |
| Semua informasi yang diperlukan untuk keputusan diagnostik dan pengobatan secara rutin tersedia bagi saya. | | | | | | | | | | | | | | | | A | | | B | | C | | D | E | | |  |
| Para peserta pelatihan dalam disiplin ilmu saya diawasi dengan cukup. | | | | | | | | | | | | | | | | A | | | B | | C | | D | E | | |  |
| Saya mengalami kolaborasi yang baik dengan perawat di bangsal ini. | | | | | | | | | | | | | | | | A | | | B | | C | | D | E | | |  |
| Saya mengalami kolaborasi yang baik dengan staf dokter di bangsal ini. | | | | | | | | | | | | | | | | A | | | B | | C | | D | E | | |  |
| Saya mengalami kolaborasi yang baik dengan staf kesehatan lain di bangsal lain | | | | | | | | | | | | | | | | A | | | B | | C | | D | E | | |  |
| Gangguan komunikasi yang menyebabkan keterlambatan dalam pemberian perawatan adalah biasa. | | | | | | | | | | | | | | | | A | | | B | | C | | D | E | | |  |

S2 Questionnaire. Indonesian version of the Safety Attitudes Questionnaire (SAQ-INA)
