# Supplementary material for: Validation of the Indonesian version of the Safety Attitudes Questionnaire: A Rasch analysis
Source: PLoS One. 2019 Apr 10;14(4):e0215128. doi: 10.1371/journal.pone.0215128 (PMC6457536; doi:10.1371/journal.pone.0215128)
Supplement: S1 Fig — (DOCX) [file pone.0215128.s003.docx]

| 1. Safety Climate   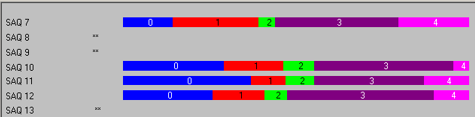 |
| --- |
| 1. Job satisfaction   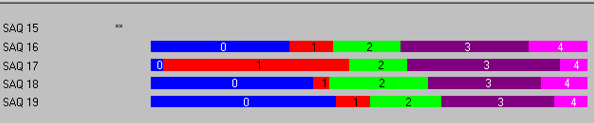 |
| 1. Stress recognition   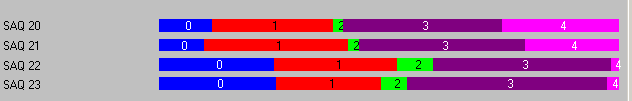 |
| 1. Perception of ward management   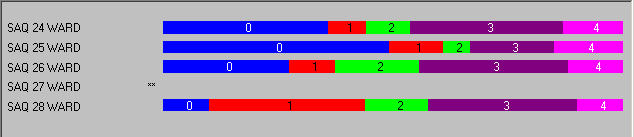 |
| 1. Perception of hospital management   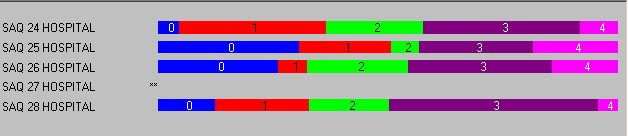 |
| 1. Working condition   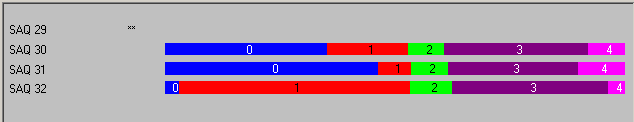  Note: Threshold map for teamwork climate domain could not be generated due to disordered threshold |

S1 Fig. Thresholds maps for the SAQ-INA domains. The numbers represent the response options of strongly disagree to strongly agree respectively for each SAQ-INA item. The length of the boxes indicates the level of respondents’ endorsement towards the response option.
