# Supplementary material for: Validation of the Indonesian version of the Safety Attitudes Questionnaire: A Rasch analysis
Source: PLoS One. 2019 Apr 10;14(4):e0215128. doi: 10.1371/journal.pone.0215128 (PMC6457536; doi:10.1371/journal.pone.0215128)
Supplement: S2 Fig — (DOCX) [file pone.0215128.s004.docx]

| a. Teamwork climate  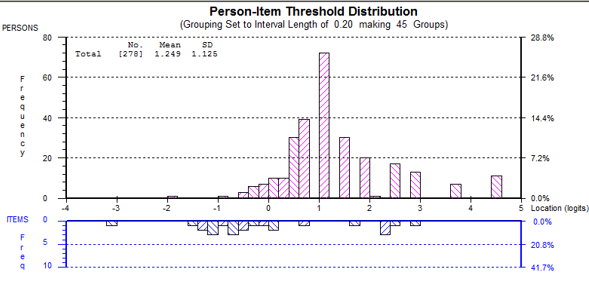 | b. Safety climate  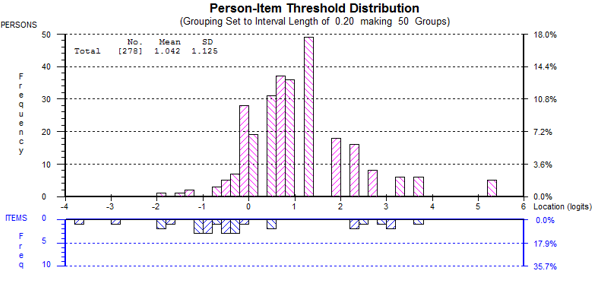 |
| --- | --- |
| c. Job satisfaction  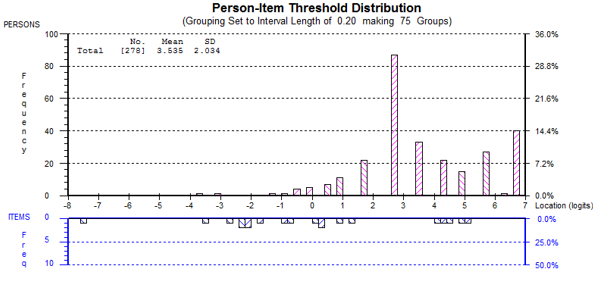 | d. Stress recognition  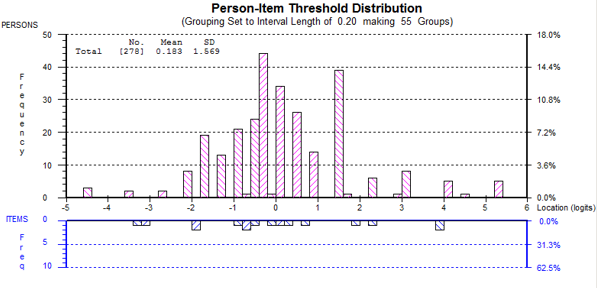 |
| e. Perception of ward management  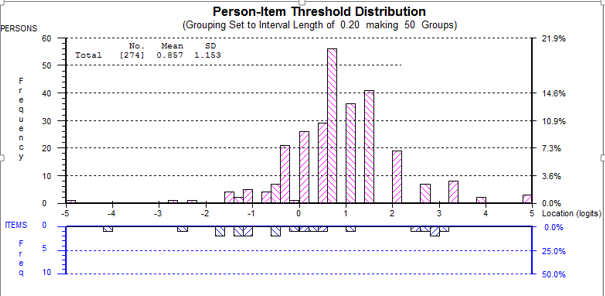 | f. Perception of hospital management  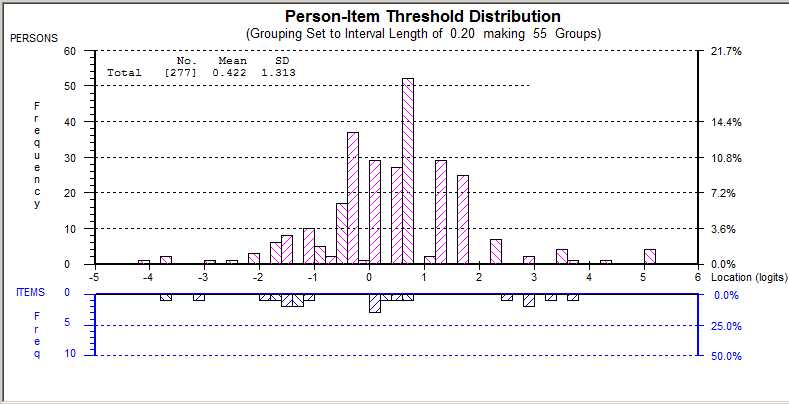 |
| g. Working conditions  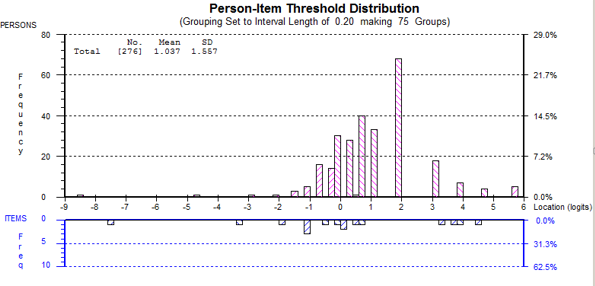 |  |

S2 Fig. Person-item thresholds distribution depicting targeting for all SAQ-INA domains. Distributions of the locations of people and items on the common logic metric where negative values refer to poor safety climate and positive values indicate good safety climate are shown on the upper and lower panels respectively.
